# Dealing with the impact of pregnancy on the pharmacokinetics of aripiprazole once-monthly

**DOI:** 10.1007/s00737-026-01678-2

**Published:** 2026-01-30

**Authors:** Georgios Schoretsanitis, Caitriona Obermann, Michael Paulzen

**Affiliations:** 1 Department of Adult Psychiatry and Psychotherapy, Psychiatric Hospital, University of Zurich, Zurich, Switzerland; 2 The Zucker Hillside Hospital, Department of Psychiatry, Northwell Health, Glen Oaks, NY USA; 3 Unit of Pharmacogenetics and Clinical Psychopharmacology, Centre for Psychiatric Neuroscience, Lausanne University Hospital, University of Lausanne, Prilly, Switzerland; 4 Alexianer Krankenhaus Aachen, Aachen, Germany

**Keywords:** Pregnancy, long-acting injectable antipsychotics, Aripiprazole once-monthly, schizoaffective disorders, therapeutic drug monitoring

Various physiological changes in different organ systems in the course of pregnancy can essentially affect the disposition of all medications and not only psychotropic medications (Westin et al. 2018a, b). Pathways underlying the impact of pregnancy on drug disposition may include changes in protein binding, hepatic and other enzymes’ activity, increases in kidney function, changes in the distribution volume and efflux transporters (Schoretsanitis et al. 2020). These changes usually progress throughout pregnancy and peak within the 3rd trimester (Schoretsanitis et al. 2020, 2024). There is only one cohort of antipsychotic-treated pregnant women with antipsychotic drug levels extracted from healthcare routine records (Westin et al. 2018a); Westin and colleagues suggested a decline of serum levels of quetiapine and oral aripiprazole up to -75% and − 52% respectively in the 3rd trimester. Here we report the case of a pregnant woman suffering from schizophrenia, in which the aripiprazole long-acting injectable (LAI) treatment was augmented with oral aripiprazole during pregnancy to offset pharmacokinetic changes related to the physiology of pregnancy.

Mrs. A., a 38-year-old female, with paranoid schizophrenia (ICD-10 F20.0), mother of two, has been treated with deltoidal injections of aripiprazole once-monthly 400 mg, to which she responded well over a prolonged time period, although she complained for mild symptoms towards the end of the injection interval. Accordingly, the aripiprazole LAI injection interval was shortened to 25 days. Trough levels for aripiprazole active moiety levels were occasionally measured with a total of four assessments over two years before the third pregnancy ranging between 497.8 and 847.5 ng/mL (Table 1). Aripiprazole active moiety levels were quantified using a validated liquid chromatography before the next LAI injection at the same injection site. The patient had signed general consent.

Upon the beginning of the third pregnancy the patient persistently asked for a shortening of the LAI injection interval to every three weeks, as she was worried about the relapse risk during pregnancy. Subsequently, an injection interval of every three weeks was established. At gestational week 18 she reported emerging symptoms, including difficulty in falling asleep, nonspecific anxiety and restlessness. Gradually she developed jealousy-related ideas concerning her husband, a well-known symptom from previous episodes. An augmentation with oral aripiprazole, 10 mg/day, was initiated in week 18. At that timepoint aripiprazole active moiety levels reached the minimum value throughout pregnancy (363.2 ng/mL). Despite short-term improvement, the positive symptoms persisted so that an oral aripiprazole dose increase up to 15 mg/day was initiated in gestational week 20. Subsequently, the patient was stable, although aripiprazole active moiety levels were in rather low range (394.6-511.2 ng/mL). During the 3rd trimester, the patient again reported positive symptoms, whereupon oral aripiprazole was increased up to 20 mg/day at gestational week 35. Hereafter she remained stable till delivery. The birth was without complications. A light green colored amniotic fluid may have indicated mild fetal stress, but Apgar scores and pH-value showed good adaptation. The first postpartum assessment of active moiety levels (914.8 ng/mL) 4 days after birth did not take place at trough-level conditions for LAI, as the assessment was performed two weeks post-injection. A further increase of levels was observed in the next assessment, 17 days after birth (1,157.0 ng/mL), whereupon the oral dose was discontinued. At no timepoint did the patient report any adverse effects related to the aripiprazole LAI or the titration. As the patient immediately stopped breastfeeding, we cannot make any statements regarding the risk of lactation failure, previously reported for aripiprazole, and/or potential dose-dependent patterns (Spann et al. 2025).

This is the first case report showing pharmacokinetic changes during pregnancy in an aripiprazole LAI-treated patient. Oral aripiprazole was added to prevent a further drop in aripiprazole concentrations, which subsequently increased accordingly and the patient responded well. Several factors may underlie the pharmacokinetic changes for LAI during pregnancy; the increase of ratios of the active metabolite divided by the parent compound during pregnancy (0.44–0.52) compared to preconception (0.22–0.37) imply an increased cytochrome P450 3A4 activity, which is mainly involved in the hepatic metabolism of aripiprazole LAI, normalizing at postpartum, when ratios dropped again (028-0.37). Besides, a mild increase of renal function may have also played a role. In our case, it is hard to quantify the impact of pregnancy solely on the pharmacokinetics of aripiprazole LAI, as we added oral aripiprazole to treat the emerging positive symptoms. However, we suggest that when psychopathology changes require adjustments in therapeutic regimens, the use of TDM can provide orientation to avoid subtherapeutic drug levels and to minimize a related relapse risk.

**Table 1:** Aripiprazole active moiety levels (ng/mL) and metabolic ratios throughout pregnancy, at preconception and at postpartum

Timepoint	LAI dose (mg)	Oral dose (mg/day)	ARI (ng/mL)	D-ARI (ng/mL)	Active moiety levels (ng/mL)^a^	Metabolic ratio^b^
109 weeks before pregnancy	400	-	363	135	498	0.37
83 weeks before pregnancy		-	439	148	587	0.34
46 weeks before pregnancy		-	646	202	848	0.31
20 weeks before pregnancy		-	614	135	749	0.22
GW 13	400	-	413	188	601	0.46
GW 16		-	251	112	363	0.45
GW 19		10^c^	350	157	507	0.45
GW 22		15^d^	350	161	511	0.46
GW 25		15	350	157	507	0.45
GW 28		15	269	139	408	0.52
GW 31		15	274	121	395	0.44
GW 34		15	341	166	507	0.49
GW 37		20^e^	309	143	452	0.46
PPW 1	400	20	668^f^	247^f^	915^f^	0.37^f^
PPW 4		-	906	251	1157	0.28
PPW 6		-	552	211	763	0.38

*ARI* aripiprazole (parent compound); *D-ARI* dehydroaripiprazole; *GW* gestational week; *PPW* week at postpartum

^a^Active moiety refers to the sum of aripiprazole parent compound and active metabolite (dehydroaripiprazole)

^b^The metabolic ratio is the ratio of the active metabolite (dehydroaripiprazole) divided by the parent compound

^c^Started at gestational week 18

^d^Oral dose increased up to 15 mg/day on gestational week 20

^e^Oral dose increased up to 20 mg/day on gestational week 35

^f^This value was not trough as the sample was drawn two weeks after the last injection

## Data Availability

No datasets were generated or analysed during the current study.
